# Energy Absorption Mechanism and Its Influencing Factors for Circular Concrete-Filled Steel Tubular Members Subjected to Lateral Impact

**DOI:** 10.3390/ma14164652

**Published:** 2021-08-18

**Authors:** Luming Wang, Yanhui Liu, Lang Yang, Nan Xu, Shichun Zhao

**Affiliations:** School of Civil Engineering, Southwest Jiaotong University, Chengdu 610031, China; lmwang@my.swjtu.edu.cn (L.W.); lyang@my.swjtu.edu.cn (L.Y.); nxu@my.swjtu.edu.cn (N.X.); zhaosc1961@163.com (S.Z.)

**Keywords:** concrete-filled steel tube (CFST), lateral impact, material strain rate, segmented numerical model, energy absorption mechanism, influencing factor

## Abstract

The energy absorption characteristic of steel tube material and concrete material is an important indicator to reflect the impact resistance of circular concrete-filled steel tubular (CFST) members. In order to efficiently simulate the material energy absorption of the steel tube and concrete under lateral impact, a nonlinear finite element model considering the material strain rate of the circular CFST member was established and validated based on the drop weight tests. Then, the energy absorption mechanism of circular CFST members subjected to lateral impact was investigated including the revelation of the energy absorption process and the determination of the energy absorption distribution for the steel tube material and concrete material, which are obtained respectively based on the comprehensive analysis of dynamic response and innovative establishment of the segmented numerical model. In addition, the influence of impact momentum on energy absorption process and the effect of impact location on energy absorption distribution are further carried out. The observations of this investigation can provide reference for the anti-impact design and damage reinforcement of circular CFST members subjected to lateral impact.

## 1. Introduction

Concrete-filled steel tubular (CFST) members exhibit advantages of high strength and favorable ductility because of the collaborative force of steel tube and core concrete, which are widely applied to high-rise and long-span engineering structures consequently [1,2]. Apart from the conventional static loads, CFST members are also threatened by accidental dynamic loads such as vehicle impact, train derailment, and rockfall impact during the whole service life cycle. It is widely known that CFST members are often utilized as the key components of engineering structures, and a serious engineering accident may happen due to the damage or failure of the CFST member caused by impact loads. Furthermore, the material energy absorption of steel tube and concrete is an important indicator to reflect the dynamic response of CFST members. Therefore, in order to enhance the impact resistance of CFST members, it is of realistic significance to investigate the energy absorption mechanism of CFST members.

Some researchers have carried out a series of investigations on the dynamic response of impacted CFST members. Deng et al. [3,4] investigated the impact resistance of circular CFST members with different spans based on the drop weight tests. Wang et al. [5] conducted drop weight tests on circular CFST members under various impacts of kinetic energy, and investigated the effects of steel tube thickness and axial force on the dynamic response. Furthermore, Aghdamy et al. [6] analyzed the sensitivity of member parameters and load parameters to the dynamic response of circular CFST members through the numerical simulation. On this basis, Wang et al. [7] investigated the impact response and proposed a deflection calculation method for axially loaded circular CFST members subjected to lateral impact. Yousuf et al. [8,9,10] studied the dynamic response and impact resistance of square CFST members at different impact locations based on impact tests and numerical simulation. Bambach et al. [11,12] proposed a calculation model of energy absorption through the lateral impact tests of CFST members. Qu et al. [13] and Han et al. [14] established a simplified analysis model of CFST members combined with numerical simulation, which can be utilized to calculate the maximum deflection and bending capacity of CFST members subjected to lateral impact. Wang et al. [15] carried out an experimental research on circular CFST columns under lateral impact at the bottom, and put forward a simplified calculation method of impact resistance based on the energy absorption. Zhu et al. [16] presented an experimental and numerical study on the impact resistance of rectangular hollow steel tubular specimens and partially concrete-filled steel tubular columns under lateral impact load, and analyzed the response of typical impact force, displacement, and strain as well as the failure mode of the specimens. Hou et al. [17] numerically investigated the response of deteriorated CFST columns subject to lateral impact to explore the life cycle of the mechanical performance under the coupled effects including the failure modes, the full-range load-displacement relationship, and the residual compressive strength. Zhao et al. [18] conducted an experimental and numerical study on the impact performance of circular concrete-filled double skin steel tubular members with an external stainless steel tube. The testing parameters were the impact height, hollowness ratio, and axial load level, then the effects of the external steel tube type (stainless steel and carbon steel) on the impact resistance were also investigated. Xian et al. [19] analyzed the lateral impact performances of square steel-reinforced concrete-filled steel tubular members by drop weight tests, and the damage evolution, sectional stress, energy variation, and energy absorption distribution were discussed through the validated numerical models. In addition, Xian et al. [20] also conducted an experimental study and finite element analysis on the lateral impact response of circular concrete-filled double-tube members. A total of 12 specimens with various parameters were tested by utilizing a drop hammer impact system, and the damage modes, impact forces, displacement responses, and energy absorption capacities of the members from the impact tests were evaluated and compared. In general, the existing investigations above-mentioned have clarified the dynamic response and its simplified calculation method of impacted CFST members, but the energy absorption mechanism of circular CFST members under lateral impact has not been deeply investigated.

In this investigation, a comprehensive literature survey on impact tests of CFST members has been conducted in which the experiment information of CFST members with local damage is shown in Table 1. It can be found that the lateral impacted CFST members had obvious local response such as local depression or local buckling in addition to the global deformation. Therefore, the energy absorption of CFST members in the whole impact process includes the energy absorption of global deformation and local damage. In [21,22,23], the energy absorption caused by global deformation of CFST members was calculated by integrating the impact force–displacement curves measured by impact tests, and in [24,25], the energy absorption caused by local damage of CFST members was calculated based on contact mechanics theory. These investigations provide a method for the calculation of global and local energy absorption, but the mechanism of global and local energy absorption across the whole impact process for circular CFST members has not been identified, and in particular, the research on the process of energy absorption from the local deformation to the global response is not clear enough. In addition, it can be seen from Table 1 that the local damage of CFST members was mainly concentrated in the impact location and support areas, which indicates that the energy absorption in these areas was relatively large. However, the distribution of energy absorption along the length for circular CFST members subjected to lateral impact has not been explored in the existing research.

To address the issues described above, a nonlinear finite element model of the circular CFST member was established based on drop weight tests, which takes into account the effects of material strain rate on dynamic response, and the accuracy of numerical simulation was validated through comparison with the test results. Then, the energy absorption mechanism of circular CFST members subjected to lateral impact was investigated numerically. On one hand, the process of energy absorption is studied by comprehensively analyzing the impact dynamic response of circular CFST members, and the characteristics of energy absorption for steel tube material and concrete material are explored in the meantime. On the other hand, the energy absorption distribution of circular CFST members is determined by creatively proposing the segmented numerical model, and the main areas of energy absorption for steel tube material and concrete material are defined at the same time. On this basis, the influence of impact momentum on the energy absorption process and the effect of impact location on energy absorption distribution were further carried out respectively, and the interaction between these factors and the material energy absorption of the steel tube and concrete is elaborated in detail. Through the observations of this investigation, it is expected to provide the basis and reference for the anti-impact design and damage reinforcement of circular CFST members subjected to lateral impact.

## 2. Numerical Simulation

### 2.1. Experiment Overview

**Figure 1:**
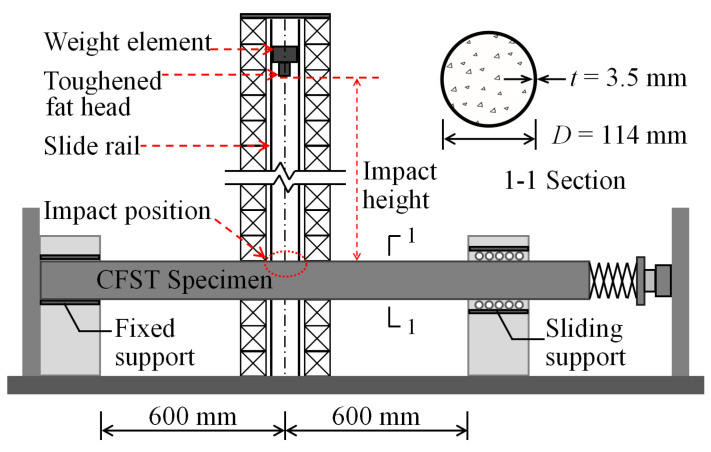
Schematic diagram of drop hammer test device.

The authors in [5] conducted a series of impact tests on circular CFST specimens through a drop hammer test machine, as shown in Figure 1. The mid-span location of the specimen was impacted vertically by the drop hammer that fell freely along the slipway during the tests. The mass of the drop hammer was 229.8 kg and the preset impact kinetic energy was achieved by changing the impact height of the drop hammer. The rigid device of the support restraint could provide the fixed-sliding boundary conditions for the circular CFST specimens. The effective length, outer sectional diameter, and steel tube thickness of the circular CFST specimens were 1200 mm, 114 mm, and 3.5 mm, respectively. The cube strength of concrete was 48.7 MPa, and the yield strength and Young’s modulus of steel tube were 298.0 MPa and 201.0 GPa, respectively. The final damage mode, impact force, and mid-span displacement of circular CFST specimens could be obtained through the drop weight tests, and the force mechanism of the whole impact process could be identified through the analysis of these dynamic responses.

### 2.2. Finite Element Model

**Figure 2:**
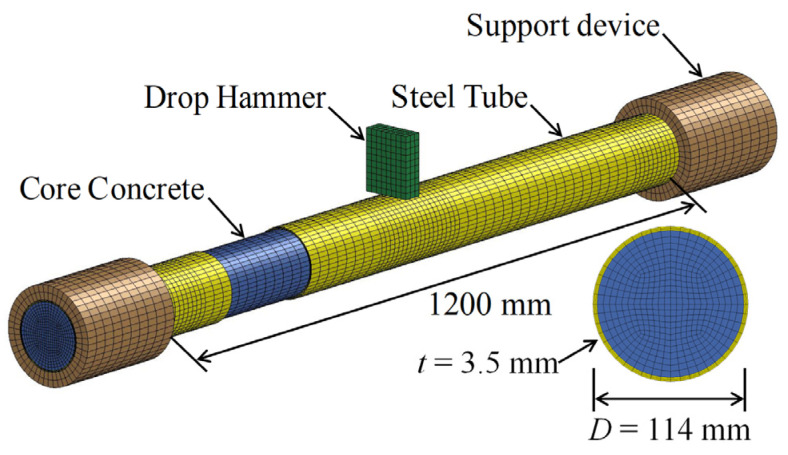
Finite element model of circular CFST member.

According to the testing principle and specimen parameters described above, the finite element model of the circular CFST member was established by utilizing the numerical simulation software LS-DYNA [26], as shown in Figure 2. The steel tube, core concrete, drop hammer, and support restraint device were simulated by employing eight-node solid elements with a single point integral (SOLID164). In order to improve the accuracy and efficiency of the simulation, the mesh was encrypted at the impact location and the support areas. The device of support restraint was simulated by the hollow cylindrical sleeves with the rigid material, which can achieve fixed-sliding boundary constraints. The drop hammer was modeled as a rigid block with the size of 30 mm × 80 mm × 100 mm, and the preset mass of the drop hammer can be obtained by adjusting the material density defined in the keyword “* MAT RIGID”. In addition, the initial velocity of the drop hammer can be set up by the keyword “* INITIAL VELOCITY GENERATION”, and the movement direction of the drop hammer was set to the vertical direction by limiting the displacement and rotation in other directions. There was no obvious slip between the steel tube and concrete in the drop weight tests, hence, the contact surface of the steel tube and concrete can be set as the common node. The contact relationship between the drop hammer, the support restraint device, and the circular CFST member can be defined by the keyword “* CONTACT AUTOMATIC SURFACE TO SURFACE”.

It is well known that steel and concrete are strain rate sensitive materials, and their apparent strength may increase significantly at high strain rates, thus the strain rate effects of materials should be considered when the CFST members are subjected to lateral impact [27]. The material model “MAT PLASTIC KINEMATIC” with the characteristics of elastic-perfectly plastic was adopted to simulate the steel tube, which can well reflect the isotropic and kinematic hardening plasticity of the steel material. The Cowper–Symonds equation [28] was utilized to reflect the strain rate effects of the steel material, and the dynamic increase factor (DIF) for the yield strength of steel under impact loads is shown in Equation (1):

(1) $$\mathrm{DIFs}=f_{\mathrm{yd}}/f_{\mathrm{ys}}=1+{\left(\dot{\varepsilon }/C\right)}^{1/P}$$

where *f*_yd_ is the dynamic yield strength of steel at the strain rate $\dot{\varepsilon }$; *f*_ys_ is the static yield strength of steel; and *C* = 6844 s^−1^ and *p* = 3.91 are the strain rate coefficients of steel [29].

The concrete was simulated by the material model “MAT CONCRETE DAMAGE REL3”, which can reflect the elastic–plastic behavior and damage failure behavior of concrete, and the keyword “* MAT ADD EROSION” was utilized to delete the failure element to avoid the excessive distortion of concrete. The dynamic increase factor (DIF) for the compressive strength of concrete recommended by the Comite Euro-international du Beton [30] is available to estimate the strain rate effects on concrete material properties, and the CDIF is expressed in Equation (2):

(2) $$\mathrm{CDIF}=\frac{f_{\mathrm{cd}}}{f_{\mathrm{cs}}}=\left\{ \begin{array}{l} {\left(\dot{\varepsilon }/{\dot{\varepsilon }}_{s}\right)}^{1.026\alpha },\;\dot{\varepsilon }\leq 30s^{-1}\\ \gamma {\left(\dot{\varepsilon }/{\dot{\varepsilon }}_{s}\right)}^{1/3},\;\dot{\varepsilon }>30s^{-1} \end{array}\right.$$

where *f*_cd_ is the dynamic compressive strength at the strain rate $\dot{\varepsilon }$; *f*_cs_ is the static compressive strength at the strain rate ${\dot{\varepsilon }}_{s}=30\times 10^{-6}{\;s}^{-1}$; and logγ=6.156α−2, in which $\alpha ={\left(5+9f_{\mathrm{cs}}/f_{\mathrm{co}}\right)}^{-1}$ and *f*_co_ = 10 MPa.

The DIF for the tensile strength of concrete was modified by Malvar [31], and the TDIF is expressed in Equation (3):

(3) $$\mathrm{TDIF}=\frac{f_{\mathrm{td}}}{f_{\mathrm{ts}}}=\left\{ \begin{array}{l} {\left(\dot{\varepsilon }/{\dot{\varepsilon }}_{s}\right)}^{\delta },\;\dot{\varepsilon }\leq 1s^{-1}\\ \beta {\left(\dot{\varepsilon }/{\dot{\varepsilon }}_{s}\right)}^{1/3},\;\dot{\varepsilon }>1s^{-1} \end{array}\right.$$

where *f*_td_ is the dynamic tensile strength at the strain rate $\dot{\varepsilon }$; *f*_ts_ is the static tensile strength at the strain rate ${\dot{\varepsilon }}_{s}=10^{-6}{\;s}^{-1}$; and logβ=6δ−2, in which $\delta ={\left(1+8f_{\mathrm{cs}}/f_{\mathrm{co}}\right)}^{-1}$ and *f*_co_ = 10 MPa.

### 2.3. Simulation Validation

**Figure 3:**
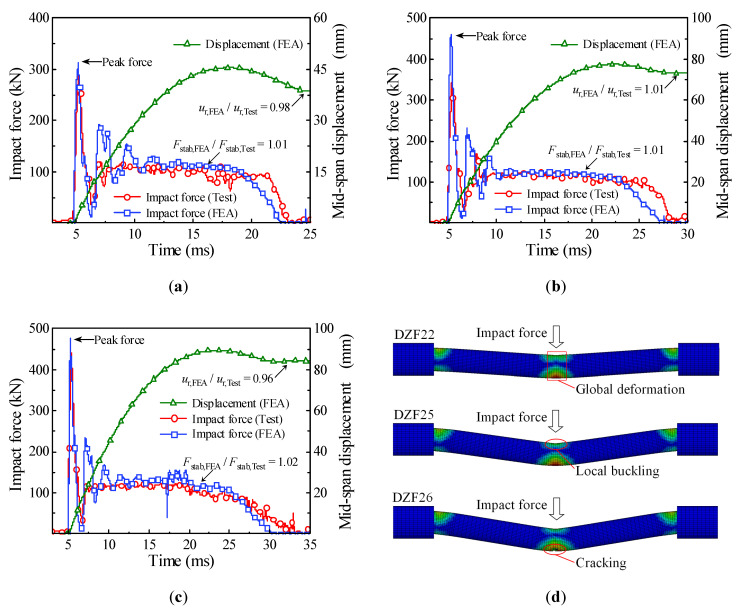
Comparison of simulation results and test results. (**a**) DZF22; (**b**) DZF25; (**c**) DZF26; (**d**) damage modes.

In order to validate the accuracy of numerical simulation, three of the impact tests present in [5] were calculated through the finite element model established above, and then the accuracy of finite element analysis was validated by comparing the simulation results of the impact force, the mid-span displacement, and the final damage mode with the test results. The comparison between the simulation results and test results is shown in Table 2 and Figure 3. It can be seen from Figure 3a–c that the time history curves of impact force obtained by numerical simulation and impact test had a consistent trend, which will go through the peak stage, stability stage, and unloading stage, and the mid-span displacement gradually decreases to the residual displacement after increasing to the maximum. It can be seen from Table 2 that the deviations of numerical simulation for platform value of impact force *F*_stab_, duration of impact force *t*_d_, and residual displacement at the impact location *u*_r_ were all kept within 5%. In addition, the damage modes of circular CFST members obtained by numerical simulation were consistent with the drop weight tests, as shown in Figure 3d, and the damage mode changed from local buckling or global deformation to cracking at the impact location with the increase in impact energy.

In conclusion, the finite element analysis method utilized in this investigation can well simulate the dynamic response of impacted circular CFST members, which indicates that the proposed numerical model and the defined material constitutive have a high accuracy. Therefore, the energy absorption mechanism of circular CFST members under lateral impact will be investigated numerically based on the validated finite element model in this paper including the revelation of energy absorption process and the determination of energy absorption distribution.

## 3. Energy Absorption Process

In order to identify the energy absorption process in detail, a typical circular CFST member was numerically simulated based on the validated finite element model established above, and the design parameters of the typical circular CFST member were reasonably set as: effective length, outer sectional diameter, and steel tube thickness of 1200 mm, 114 mm, and 3.0 mm, respectively; the cube strength of concrete was 50 MPa and the yield strength of steel tube was 360 MPa; the boundary constraint and impact location were fixed-sliding and mid-span, respectively; and the impact mass and impact velocity of the drop hammer were 50.0 kg and 24.0 m/s, respectively.

**Figure 4:**
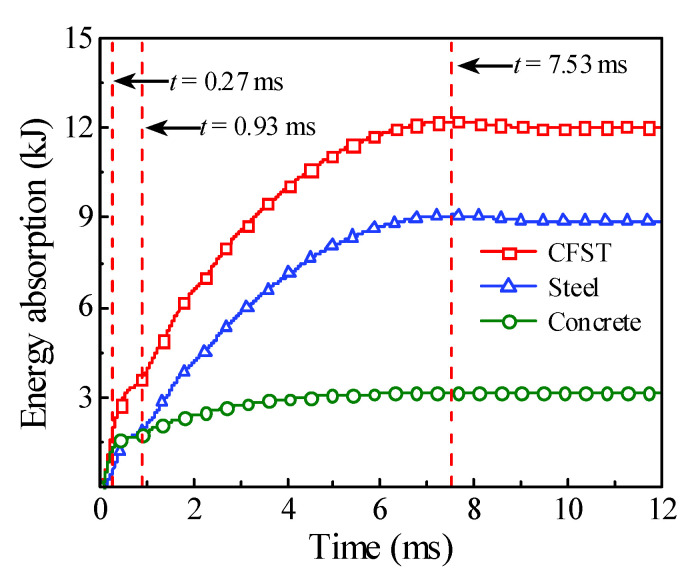
Energy absorption versus time curve.

**Figure 5:**
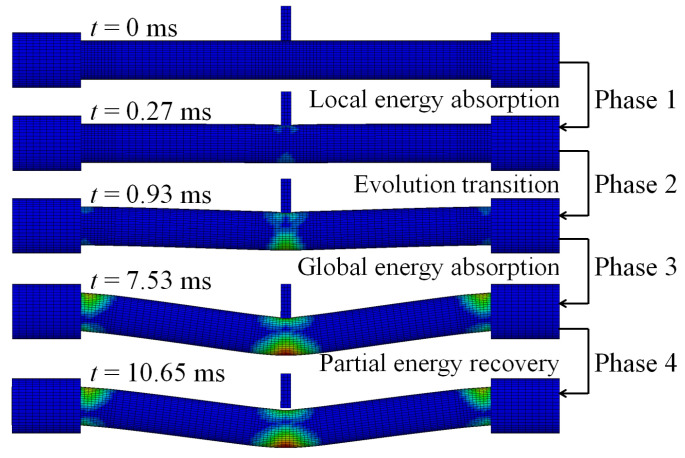
Evolution of effective plastic strain.

**Figure 6:**
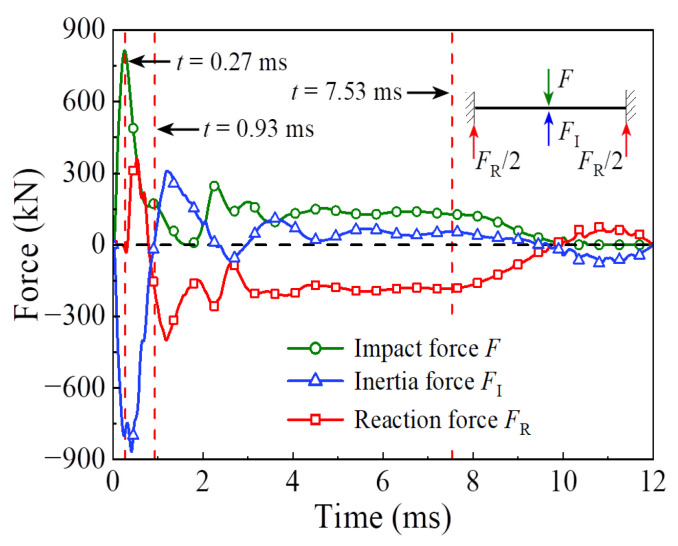
External forces versus time curve.

**Figure 7:**
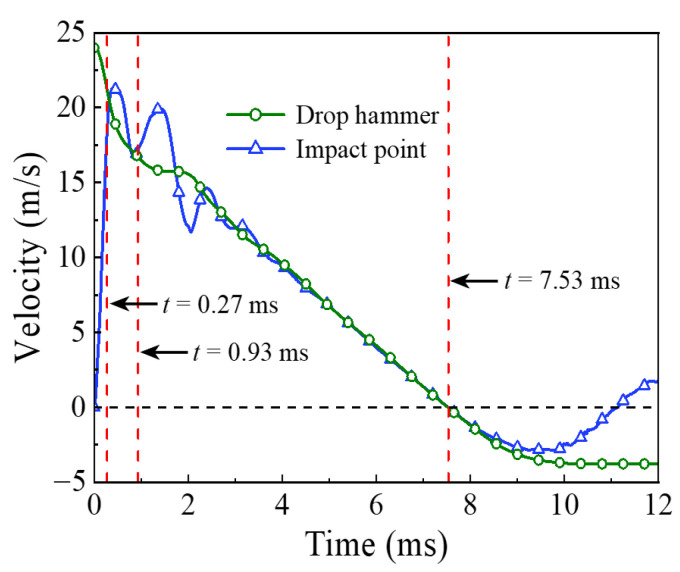
Velocity versus time curve.

**Figure 8:**
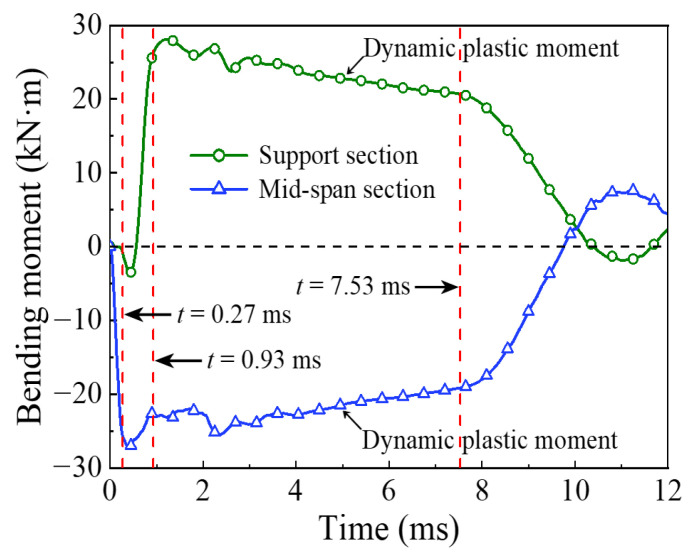
Bending moment versus time curve.

**Figure 9:**
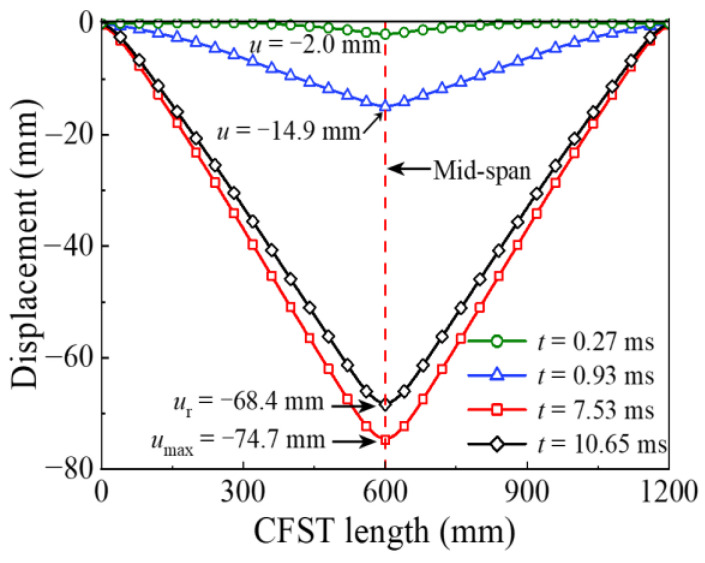
Deformation curve at different moments.

The energy absorption of the steel tube material, concrete material, and integral member during the whole impact process is shown in Figure 4. Since the impact kinetic energy is transformed into the internal energy of the circular CFST member through the strain energy generated by elements, the energy absorption process can be reflected from the evolution of plastic strain for the circular CFST member, as shown in Figure 5. Meanwhile, combined with the comprehensive analysis of the dynamic response (force time history curve shown in Figure 6, velocity time history curve shown in Figure 7, moment time history curve shown in Figure 8, and deformation curve shown in Figure 9), the energy absorption process of the circular CFST member can be reasonably divided into the phase of local energy absorption, the transition phase of energy absorption, the phase of global energy absorption, and the phase of partial energy recovery.

### 3.1. Phase 1: Local Energy Absorption

The phase of local energy absorption starts from the moment when the drop hammer contacts with the circular CFST member to the moment when the plastic strain first appears at the bottom of the mid-span. In this phase, the velocity of impact point increases rapidly to be equal to that of the drop hammer, resulting in an instantaneous increase in the impact force, the inertia force, and the bending moment of the mid-span section. However, since the stress wave has not propagated to the support position yet, the reaction force and the bending moment of support section remain at zero, and the circular CFST member only slightly displaces near the impact location during this phase.

In summary, it can be concluded that the dynamic response of the circular CFST member in the phase of local energy absorption mainly manifested in the local deformation at the impact location. For the circular CFST member simulated in this investigation, the energy absorption of Phase 1 accounted for 13.8% of the maximum energy absorption in the whole impact process, thus the effect of the local dynamic response and the local energy absorption should be fully recognized in an anti-impact design.

In addition, it was observed from this numerical investigation that the core concrete at the impact location exhibited obvious plastic deformation under the action of extrusion caused by local buckling of the steel tube, and it was the same as the experimental phenomenon in [14,22] where the core concrete was crushed at the impact location, which indicates that the energy absorption of the concrete material is significant in the impact location. For the circular CFST member simulated in this investigation, the energy absorption of concrete in the phase of local energy absorption was calculated, which accounted for 72.8% of the total energy absorption in this phase. This is enough to demonstrate that the concrete material exhibits a more important role than the steel tube material in the phase of local energy absorption.

### 3.2. Phase 2: Energy Absorption Transition

In the transition phase of energy absorption, the plastic strain of the circular CFST member not only developed further at the bottom of the mid-span, but also began to appear near the supports. In this phase, the impact force and the inertia force gradually decreased from the peak value, and the reaction force and the bending moment of support section began to form and increase significantly. As a result, the deformation pattern of the circular CFST member correspondingly evolved from local depression at the impact location to global displacement along the length of the circular CFST member.

To sum up, it can be concluded that the energy absorption pattern of the circular CFST member gradually evolved from local energy absorption to global energy absorption in the transition phase of energy absorption. For the circular CFST member simulated in this investigation, the energy absorption of Phase 2 accounted for 16.0% of the maximum energy absorption in the whole impact process, and the steel tube exhibited a higher energy absorption capacity than that of concrete in this phase.

### 3.3. Phase 3: Global Energy Absorption

In the phase of global energy absorption, the plastic strain at the impact location and near the supports of the circular CFST member has developed to a great extent. In this phase, the velocity of the impact point fluctuated briefly, and then remained consistent with the velocity of the drop hammer and decreased to zero in coordination. In the meantime, the impact force, inertia force, and reaction force gradually tended to be stable after several rounds of vibration, and the bending moments of the mid-span section and the support section were also in a stable process as a whole. Due to the relatively long duration of external loads and relatively stable variation of internal forces in this phase, the circular CFST member exhibited the characteristics of global dynamic response, and the global displacement along the length of itself gradually reached the maximum deformation state.

Taken together, it can be concluded that the circular CFST member presented a large degree of global deformation performance and a high level of energy absorption capacity in the phase of global energy absorption. For the circular CFST member simulated in this investigation, the energy absorption of Phase 3 accounted for 70.2% of the maximum energy absorption of the whole impact process. Thus, it can be demonstrated that the phase of global energy absorption is the main process of energy absorption. It is worth noting that the steel tube material exerted a strong energy absorption capacity, and its energy absorbed was 5.1 times that of concrete material in this phase. Therefore, it is advisable to improve the energy absorption performance of circular CFST members by appropriately enhancing the strength and content of the steel tube in the anti-impact design.

### 3.4. Phase 4: Partial Energy Recovery

In the phase of partial energy recovery, the plastic strain at the impact location and the support areas of the circular CFST member was slightly weakened to the final state. In this phase, the circular CFST member and the drop hammer rebound slightly until the drop hammer separates from the member, hence the impact force, the inertial force and the reaction force unload gradually, and the global displacement along the length of the circular CFST member changes from the maximum deflection to the residual deformation.

Above all, it can be concluded that the part of the internal energy of the circular CFST member is converted into the kinetic energy of the drop hammer in the phase of partial energy recovery. For the circular CFST member simulated in this investigation, the internal energy decreased by 1.7% in Phase 4 compared with the maximum internal energy. It should be noted that the energy absorbed by the concrete material is basically constant in this phase, while the kinetic energy of the drop hammer is mainly provided by the internal energy released by the elastic deformation recovery of the steel tube.

## 4. Energy Absorption Distribution

**Figure 10:**
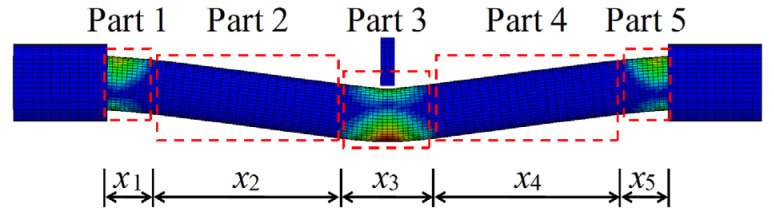
Division of energy absorption areas.

According to the damage pattern of the impacted CFST members shown in Table 1, it can be found that the damage of the CFST members was relatively serious at the impact location and the support areas, which indicates that the energy absorption distribution along the length for CFST members is non-uniform and non-linear. In order to determine the energy absorption distribution of circular CFST members subjected to lateral impact, based on the evolution and pattern of plastic strain for impacted CFST members shown in Figure 5, the energy absorption areas can be preliminarily divided into five parts, as shown in Figure 10. Then, the main areas of energy absorption can be accurately determined by changing the values of *x*_1_–*x*_5_ to analyze the energy absorption of the five parts divided in Figure 10.

**Figure 11:**
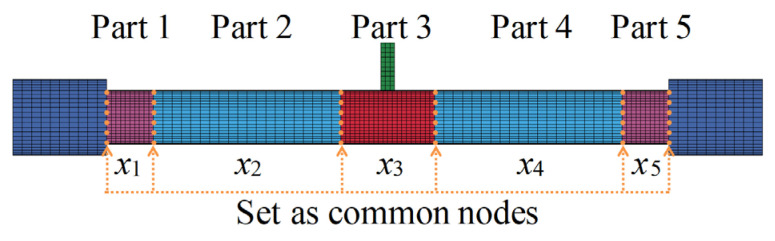
Segmented numerical model.

**Figure 12:**
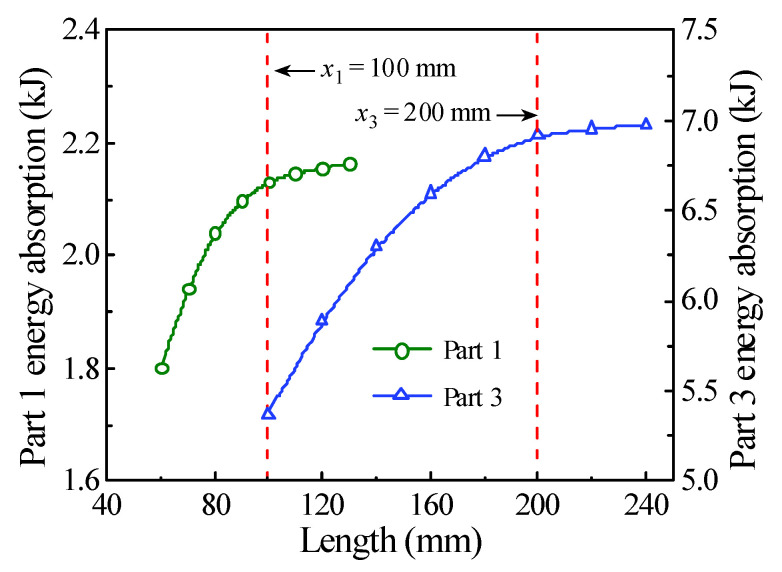
Variation of Parts 1 and 3 energy absorption.

In order to obtain the internal energy of each part of a circular CFST member, it is necessary to creatively segment the numerical model of the circular CFST member in the finite element analysis, and at the same time, the connection between each segmented part is treated with a common node to ensure the continuous deformation of the integral member. The segmented numerical model was established creatively in LS-DYNA based on the design parameters of the typical circular CFST member, as shown in Figure 11. The energy absorption distribution of the circular CFST member with the different values of *x*_1_–*x*_5_ can be obtained by extracting the internal energy of each segmented part. Figure 12 reflects the changes in energy absorption of Parts 1 and 3 with the various *x*_1_ and *x*_3_, respectively. Obviously, with the increase in *x*_3_, the energy absorption of the circular CFST member in this area also increased. When *x*_3_ was increased to 200 mm, the energy absorption at the impact location will gradually stabilize, so it can be concluded that the main area of energy absorption at the impact location was within 200 mm (i.e., 1/6 of the effective length of the circular CFST member). Similarly, the main area of energy absorption at the end of the circular CFST member was within 100 mm (i.e., 1/12 of the effective length of the circular CFST member).

**Figure 13:**
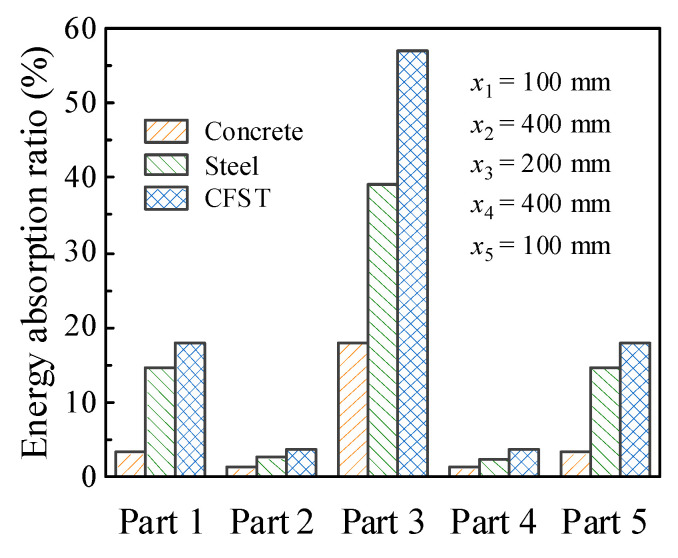
Energy absorption distribution.

**Figure 14:**
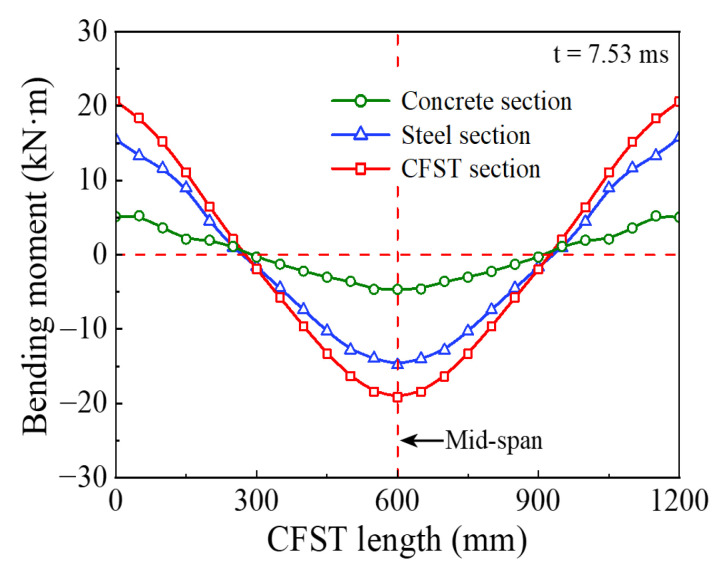
Bending moment distribution curve.

Figure 13 shows the percentage of energy absorption of each segmented part for the circular CFST member to the total energy absorption. The energy absorption of the main areas at the impact location (*x*_3_ = 200 mm) and the end of the circular CFST member (*x*_1_ = *x*_5_ = 100 mm) respectively accounted for 57.1% (Part 3) and 35.7% (Parts 1 and 5) of the total energy absorption. However, the energy absorption of the rest parts (Parts 2 and 4) of the circular CFST member only accounted for 7.2% of the total energy absorption. Thus, it can be demonstrated that the main areas of energy absorption resist most of the impact kinetic energy, and the cumulative damage is also concentrated in the main areas of energy absorption correspondingly. In addition, it can be concluded from Figure 13 that the steel tube exhibited a high energy absorption capacity at both the impact location and the end of the member, while the concrete only consumed a small part of the initial kinetic energy at the impact location, and the rest of the concrete was in a low energy absorption level, which is consistent with the distribution of bending moment for the steel tube material, concrete material, and integral member, as shown in Figure 14. Therefore, it is meaningful to pay more attention to the characteristics of energy absorption at the impact location (within 1/6 of the effective length) and the end of the member (within 1/12 of the effective length) in the anti-impact design and damage reinforcement of circular CFST members subjected to lateral impact at the mid-span.

## 5. Influencing Factors of Energy Absorption Mechanism

### 5.1. Influence of Impact Momentum on Energy Absorption Process

It is well known that the impact momentum will vary with the different combinations of impact mass and impact velocity when the impact kinetic energy remains constant, which results in the circular CFST members exhibiting various dynamic responses under lateral impact [32]. In particular, the change in impact velocity has a significant effect on the local response of structural members, which is presented in [33]. In order to investigate the influence of impact momentum on energy absorption process, a series of numerical simulations with the different impact momentum of circular CFST members were carried out when the impact kinetic energy remained constant. Five sets of impact parameters (*P*/kg·m·s^−1^, *m*/kg, *v*/m·s^−1^) in the numerical simulation were set as (800, 22.2, 36), (960, 32.0, 30), (1200, 50.0, 24), (1600, 88.8, 18), and (2400, 200, 12) based on the design parameters of the typical circular CFST member, where *P* is the impact momentum, *m* is the impact mass, and *v* is the impact velocity.

**Figure 15:**
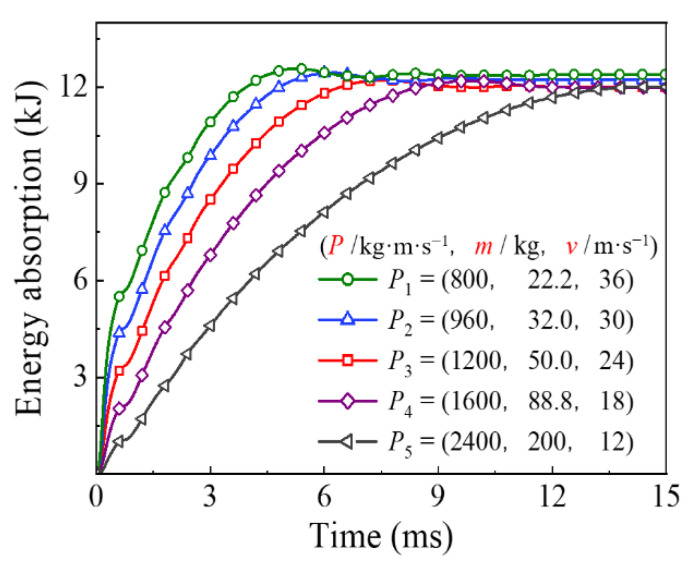
Variation of energy absorption with different impact momentum.

**Figure 16:**
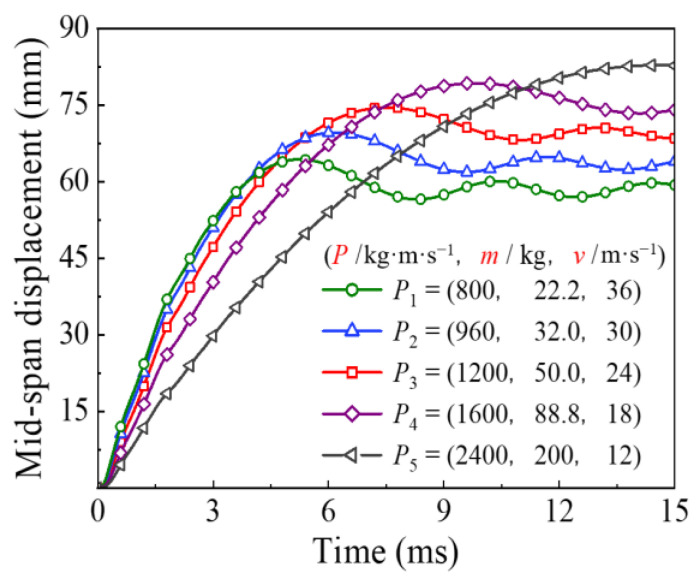
Variation of mid-span displacement with different impact momentum.

The comparison of time history for the energy absorption and the mid-span displacement under the different impact momentum are shown in Figure 15 and Figure 16, respectively. It can be seen that the change in impact momentum had little effect on the final energy absorption of the circular CFST member when the impact kinetic energy remained constant. In contrast, the energy absorption process varied greatly with the change in impact momentum, which led to a significant variation in mid-span displacement for the circular CFST member. The above changes can be mainly shown as follows: when the impact kinetic energy remains constant, the impact duration and residual deformation of the circular CFST member become larger with the increase in impact momentum.

**Figure 17:**
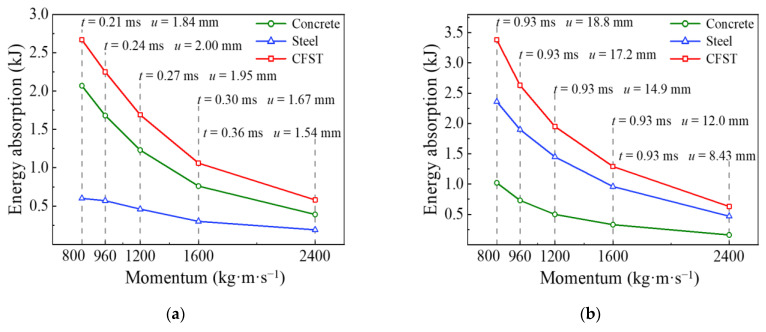

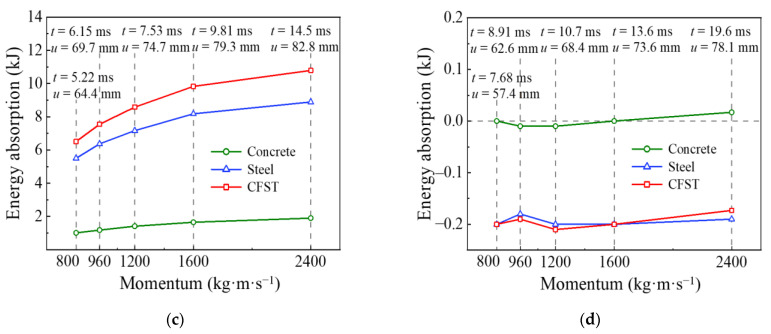
Influence of impact momentum on each energy absorption phase. (**a**) Phase 1; (**b**) Phase 2; (**c**) Phase 3; (**d**) Phase 4.

In order to investigate the influence of impact momentum on each energy absorption phase for the circular CFST member in detail, the variation curves of each energy absorption phase with impact momentum were extracted from the whole energy absorption process, as shown in Figure 17. It can be concluded from Figure 17a that the energy absorption of the circular CFST member becomes larger with the decrease in impact momentum in the phase of local energy absorption (Phase 1), which is due to the increase in impact velocity, and the decrease in impact mass led to the reduction in duration in Phase 1 and the aggravation of local damage for the circular CFST member. It is noteworthy that the concrete material showed higher energy absorption performance than the steel tube material in Phase 1, and with the decrease in impact momentum (i.e., the impact velocity increased and the impact mass decreased), the energy absorption of the concrete material increased more than that of the steel tube material, which was due to the aggravation of local depression increasing the energy absorption of the concrete material. From the above analysis, it can be seen that the concrete material exerts a relatively high energy absorption capacity in Phase 1, which has a beneficial effect on resisting the local depression caused by the larger impact velocity.

It can be concluded from Figure 17b that the end time of the circular CFST member in the transition phase of energy absorption (Phase 2) was 0.93 ms, which does not vary with the impact momentum. However, the mid-span displacement of the circular CFST member became larger with the decrease in impact momentum (i.e., the increase in impact velocity and the decrease in impact mass) in the process of stress wave transferring from the impact location to the support areas. Correspondingly, the energy absorption of the circular CFST member also increased with the reduction in impact momentum in Phase 2, and the steel tube material showed a greater energy absorption capacity than the concrete material.

It can be concluded from Figure 17c that the energy absorption and mid-span displacement of the circular CFST member becomes larger with the increase in impact momentum in the phase of global energy absorption (Phase 3). It is not difficult to explain this phenomenon in combination with the above analysis, that is, the energy absorption of the circular CFST member in Phase 1 and 2 reduces with the increase in impact momentum, thus the energy absorption in Phase 3 is bound to become larger with the increase in impact momentum under the condition that the impact kinetic energy remains constant. In addition, with the increase in impact momentum, the energy absorption of the steel tube material increased greatly in Phase 3, while that of the concrete material only increased slightly.

It can be concluded from Figure 17d that the impact momentum has little effect on the energy release of the circular CFST member in the phase of partial energy recovery (Phase 4). Since the energy release of concrete material is basically maintained at zero in Phase 4, only the energy release of steel tube material varied slightly with the change in impact momentum, which is ultimately reflected in the transformation for the internal energy of the steel tube to the kinetic energy of the drop hammer.

**Figure 18:**
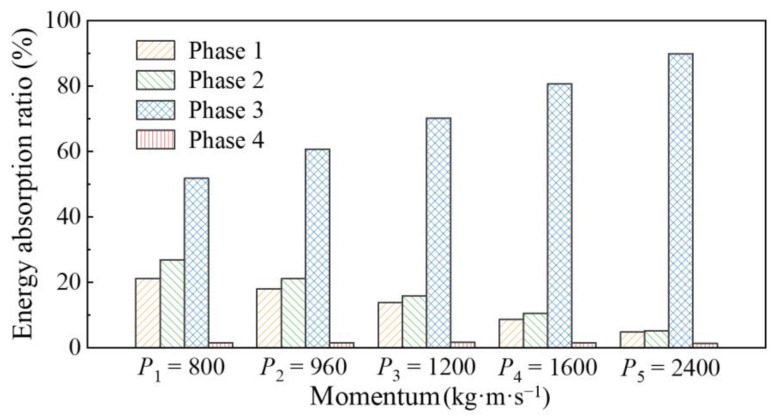
Influence of impact momentum on the energy absorption process.

In summary, the percentage of energy absorption in each phase to the total energy absorption for the circular CFST member under different impact momentum can be obtained, as shown in Figure 18, and combined with the local response and global response of the circular CFST member in each phase, the following conclusions can be drawn: (1) the energy absorption of the circular CFST member in Phase 1 reduces with the increase in impact momentum, which is mainly exhibited in the weakening of local damage for the circular CFST member; (2) the energy absorption of the circular CFST member in Phase 2 also reduces with the increase in impact momentum, which is due to the change in impact momentum affecting the transformation between the local response and the global response for the circular CFST member; (3) the energy absorption of the circular CFST member in Phase 3 becomes larger with the increase in impact momentum, which is mainly reflected in the substantial increase in the global deformation for the circular CFST member; and (4) the energy release of the circular CFST member in Phase 4 does not vary obviously with the change in impact momentum, which is ultimately manifested in the transformation for the internal energy of the steel tube to the kinetic energy of the drop hammer.

In addition, combined with the mapping relationship between the energy absorption of materials and the dynamic response of the circular CFST member, it can be concluded that the energy absorption of the steel tube material is mainly reflected in the global deformation of the circular CFST member, while the energy absorption of concrete material is mainly reflected in the local damage at the impact location.

### 5.2. Influence of Impact Location on Energy Absorption Distribution

For a circular CFST member that is impacted laterally at the mid-span, the main areas of energy absorption are symmetrically distributed at the impact location and the end of the circular CFST member, as shown in Figure 10. The authors in [8,9,10] pointed out that the dynamic response of the structural members varies with the impact location, so it is reasonable to predict that the energy absorption distribution of structural members is also affected by the impact location. In order to investigate the influence of impact location on the energy absorption distribution of circular CFST members in detail, a series of numerical simulations was carried out based on the design parameters of the typical circular CFST member, where the impact locations were set at the 1/2 span, 3/8 span, 1/4 span, and 1/8 span.

**Figure 19:**
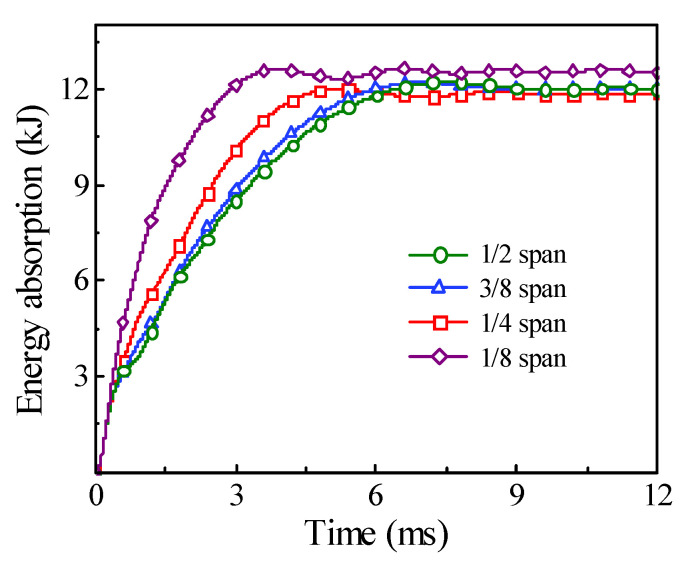
Variation of energy absorption with different impact locations.

**Figure 20:**
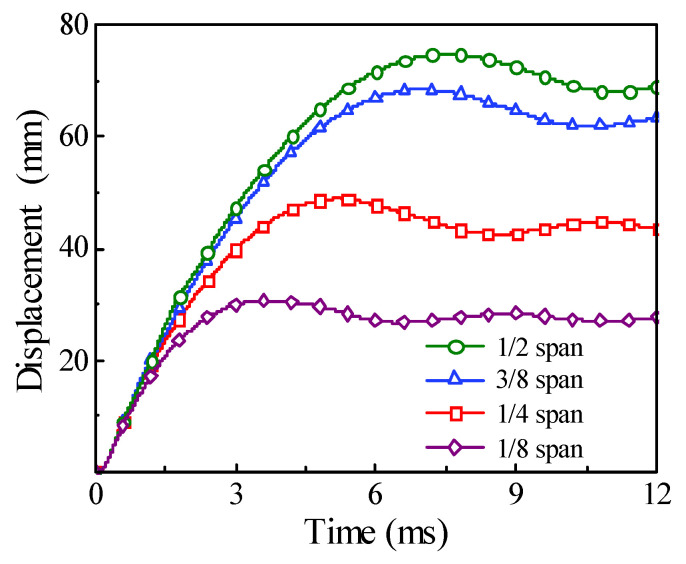
Variation of displacement with different impact locations.

The comparison of time history for the energy absorption and the displacement under the different impact locations are shown in Figure 19 and Figure 20, respectively. It can be found that, with the impact location approaching from the mid-span to one support, the final energy absorption of the circular CFST member will increase slightly, while the displacement of the impact location will decrease greatly, which indicates that the main area of energy absorption for the circular CFST member is gradually changing from the impact location to the proximal support.

**Figure 21:**
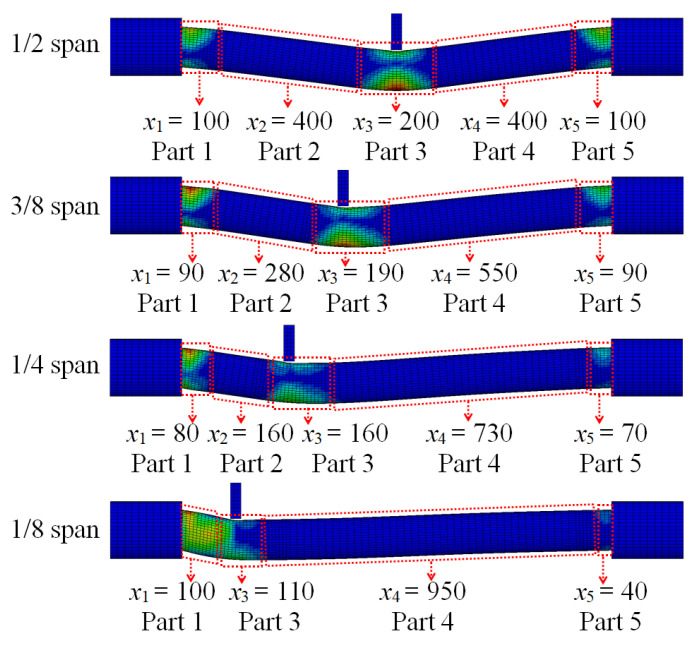
Variation of energy absorption areas under different impact locations (unit: mm).

The main areas of energy absorption for the circular CFST member under different impact location can be determined based on the segmented numerical model. As shown in Figure 21, it can be concluded that as the impact location approaches from the mid-span to one support, the main areas of energy absorption for the circular CFST member become more concentrated, and the length for the main areas of energy absorption at the impact location (Part 3) and the end of the circular CFST member (Parts 1 and 5) decreases continuously.

**Figure 22:**
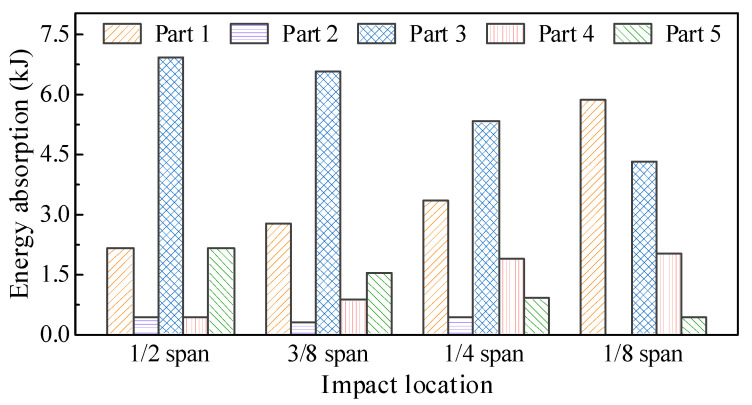
Influence of impact location on energy absorption distribution.

The energy absorption distribution of the circular CFST member under different impact locations is shown in Figure 22, which was obtained by extracting the internal energy of each segmented part (i.e., Parts 1–5 shown in Figure 21). It can be observed that with the impact location approaching from the mid-span to one support, the energy absorption at the impact location (Part 3) and the distal support (Part 5) decreased continuously, while the energy absorption at the proximal support (Part 1) increased greatly, which made the damage in the proximal support area more serious. Therefore, it is necessary to consider the difference in energy absorption distribution caused by the change in impact location in the anti-impact design and damage reinforcement for circular CFST members subjected to lateral impact.

## 6. Conclusions

A nonlinear finite element model considering the strain rate effects of the circular CFST member was established and validated in this investigation. Then, the energy absorption mechanism of circular CFST members under lateral impact was investigated including elucidating the energy absorption process and the determination of the energy absorption distribution. On this basis, the influence of impact momentum and impact location on energy absorption mechanism was further carried out in detail. From the results, the major conclusions can be summarized as follows:

1. Based on the comprehensive analysis of dynamic response, the energy absorption process can be divided into four phases, which are the phase of local energy absorption, the transition phase of energy absorption, the phase of global energy absorption, and the phase of partial energy recovery, where the phase of global energy absorption is the main process. As the main material to resist the tensile deformation, the steel tube material exhibits a perfect energy absorption performance in the whole impact process, while the concrete material mainly exerts its energy absorption capacity in the local response phase due to the local depression at the impact location.
2. The segmented numerical model was proposed creatively in this investigation to determine the energy absorption distribution of circular CFST members, and the main areas of energy absorption were distributed at the impact location and the end of the member. The steel tube material had a high energy absorption capacity at both the impact location and the end of the member, while the concrete material only consumed a small part of kinetic energy at the impact location, and the rest of the concrete material was at a low energy absorption level.
3. The impact momentum has a great influence on the energy absorption process of circular CFST members under lateral impact. Unlike the phase of local energy absorption, the increase in impact momentum will significantly enlarge the global energy absorption of circular CFST members when the impact kinetic energy remains constant. The energy absorption of the steel tube material and concrete material are mainly reflected in the global deformation and local damage of circular CFST members, respectively.
4. The change in the impact location will make an obvious difference in the energy absorption distribution for circular CFST members. With the impact location approaching from the mid-span to one end of the circular CFST member, the main area of energy absorption will be more concentrated, and its length will be reduced accordingly, which results in a significant increase in the energy absorption at the end of the member near the impact location.

## Figures and Tables

**Table 1:** Experiment information of CFST members with local damage.

Ref.	No.	*L* × *D* × *t* (mm × mm × mm)	Section Form	Impact Location	*m* (kg)	*v* (m/s)	*E*_g_/*E*_i_	Local Damage Pattern
[5]	DZF23	1200 × 114 × 3.5	Circle	*L*/2	229.8	9.8	-	Impact location depression
	DZF25	1200 × 114 × 3.5	Circle	*L*/2	229.8	10.8	-	Support area buckling
[8,9,10]	M-S	2500 × 100 × 5.0	Square	*L*/2	592.0	3.57	-	Impact location depression
	S-S	2500 × 100 × 5.0	Square	*L*/2	592.0	3.57	-	Impact location depression
[25]	CCFP-1-1	1800 × 219 × 10.0	Circle	*L*/2	1350.0	7.83	0.86	Impact location depression
	CCFP-2-1	1800 × 219 × 6.3	Circle	*L*/2	1350.0	7.59	0.94	Impact location depression
	CCFP-3-1	1800 × 219 × 5.0	Circle	*L*/2	1350.0	7.19	0.91	Impact location depression
[15]	CFST1	1300 × 300 × 3.75	Circle	4*L*/13	1580.0	7.79	0.79	Impact location buckling
	CFST3	1300 × 300 × 3.75	Circle	4*L*/13	1580.0	5.94	0.71	Impact location buckling
	CFST4	1500 × 300 × 3.75	Circle	4*L*/15	1580.0	7.66	0.88	Support area buckling
	CFST5	1500 × 300 × 3.75	Circle	4*L*/15	1580.0	5.92	0.88	Support area buckling
	CFST7	1500 × 300 × 3.75	Circle	4*L*/15	1780.0	4.87	0.76	Support area buckling
[22]	HS7-100-6	1500 × 180 × 6.0	Square	*L*/2	424.0	11.71	0.67	Impact location buckling
[23]	R-ST6	1200 × 114 × 2.0	Circle	*L*/2	206.65	9.39	-	Impact location buckling

Note: *L* is the effective length; *D* is the outer sectional diameter of circular CFST members or the cross-section length of square CFST members; *t* is the steel tube thickness; *m* is the impact mass; *v* is the impact velocity; *E*_i_ is the impact kinetic energy; *E*_g_ is the energy absorbed by global bending deformation.

**Table 2:** Numerical simulation validation.

No.	*h* (m)	*E*_i_ (kJ)	*F*_stab_ (kN)			*t*_d_ (ms)			*u*_r_ (mm)		
			Test	FEA	FEA/Test	Test	FEA	FEA/Test	Test	FEA	FEA/Test
DZF22	3.0	6.76	112.8	113.6	1.01	18.2	17.8	0.98	39.4	38.5	0.98
DZF25	6.0	13.51	125.2	126.2	1.01	25.0	23.7	0.95	72.4	73.1	1.01
DZF26	7.0	15.76	123.2	125.3	1.02	27.0	25.5	0.95	87.2	83.7	0.96

Note: *h* is the impact height of drop hammer; *E*_i_ is the impact kinetic energy.

## Data Availability

The datasets used and analyzed during the current study are available from the corresponding author on reasonable request.
